# Inside-Out Overview of the Increasing Organ Transplant Access Model: Bridging the Gap between Stakeholders, Providers, Patients, and Donors

**DOI:** 10.34067/KID.0000000780

**Published:** 2025-03-21

**Authors:** Karim M. Soliman, Prince M. Anand, Amy Perry, Wisit Cheungpasitporn, Ahmed Daoud, Ahmed I. Kamal, Tibor Fulop, Derek DuBay

**Affiliations:** 1 Division of Nephrology, Department of Medicine, Medical University of South Carolina, Charleston, South Carolina; 2 Medical Services, Ralph H. Johnson VA Medical Center, Charleston, South Carolina; 3 Division of Nephrology, Department of Medicine, Lancaster Medical Center, Medical University of South Carolina, Lancaster, South Carolina; 4 Division of Nephrology and Hypertension, Department of Medicine, Mayo Clinic, Rochester, Minnesota; 5 Division of Transplant Surgery, Prisma Health University Medical Group, Greenville, South Carolina

**Keywords:** renal transplantation

## Background

On November 26, 2024, the Biden-Harris Administration finalized the increasing organ transplant access (IOTA) model, which was officially published on December 4, 2024. Implemented by the Centers for Medicare & Medicaid Services (CMS), the model is designed to drive transformative changes in kidney transplantation practices across the United States.^1^ The model aims to increase transplant rates, enhance process transparency for transplant candidates, and prioritize long-term post-transplant outcomes.^2^ The initial IOTA proposal (2023) aimed to improve transplant outcomes by linking financial incentives to performance metrics across achievement, efficiency, and quality. A key feature was the $8000 upside payment per transplant, designed to reward centers for exceeding benchmarks in areas such as transplant volume, waitlist management, and patient survival. The proposal sought to address disparities in access, reduce organ discards, and promote transparency through standardized public reporting. However, feedback from stakeholders and American Society of Nephrology recommendations highlighted concerns about unintended consequences, such as potential gaming of the system or inequitable patient selection. This input led to refinements in the final model (2024), including safeguards to ensure ethical practices, adjustments to risk-adjustment methodologies, and enhanced transparency measures to maintain trust and equity in the transplant system. Final key features of the IOTA model are summarized in Table 1. This perspective critically examines the IOTA model, exploring its benefits, challenges, potential effect on clinical practice, patient outcomes, and health care equity, while offering recommendations for stakeholders.

**Table 1:** Summary of increasing organ transplant access model participation and incentives

Feature	Details
Participation	50% of kidney transplant programs in selected donation service areas
Eligibility criteria	Nonpediatric hospitals performing ≥11 kidney transplants annually during a 3-yr baseline
Performance period	July 1, 2025–June 30, 2031
Incentives	Upside risk: Up to $15,000 per Medicare fee-for-service transplant
	Downside risk: Maximum penalty of $2000 per FFS transplant for underperformance
Performance metrics	Achievement: 60 points
	Efficiency: 20 points
	Quality: 20 points

FFS, fee-for-service.

## Strengths of the IOTA Model

1. Enhanced incentives for transplantation: The model nearly doubles the maximum upside payment per transplant from $8000 to $15,000. This significant financial incentive acknowledges the complexities of managing medically challenging patients and those who receive high-kidney donor profile index donor organs. By aligning financial rewards with transplant growth, IOTA motivates centers to innovate and expand access for kidney transplantation, especially for underserved populations. For example, a center performing 50 transplants annually could gain up to $750,000 in upside payments, incentivizing growth. Conversely, underperformance risks penalties up to $100,000 (50 transplants×$2000), creating a high-reward, moderate-risk framework.
2. Focus on long-term outcomes: As recommended by the American Society of Nephrology, prioritizing quality metrics and multiyear outcomes over volume and short-term metrics, such as 90-day and 1-year benchmarks, promotes a more comprehensive perspective on transplant success. However, this approach may result in reduced emphasis on equity measures (*e.g*., organ offer transparency) during finalization. This change aligns well with the need to prioritize graft survival, patient quality of life, and health care sustainability over immediate postoperative results, and with overall societal benefits. Centers with superior patient and graft survival rates and low complication rates may receive higher incentive payments, while those with suboptimal outcomes may face financial penalties or reduced incentives.
3. Transparency in waitlist acceptance criteria: The requirement for centers to publicly disclose waitlist acceptance criteria increases accountability, transparency, and empowers patients to make informed decisions. It also mitigates disparities by standardizing practices across centers. Although some centers previously published criteria voluntarily, IOTA mandates this for all participants, standardizing transparency. A center that reduces wait times and improves organ acceptance rates without compromising quality may qualify for additional incentives, whereas inefficiencies in these areas could result in lower payments.
4. Simplification and reduced administrative burden: The removal of certain quality measures is expected to reduce administrative complexity and will allow transplant programs to focus resources on patient care, rather than compliance with excessive reporting requirements. However, it should be noted that the recently proposed health resources and services administration data directive for Organ Procurement and Transplantation Network data collection may negate this benefit.^3,4^
5. Alignment with Advancing American Kidney Health Initiative: The IOTA model builds on the 2019 Advancing American Kidney Health initiative, reinforcing the goals of increasing access to transplantation and promoting home dialysis. This continuity underscores a commitment to holistic ESKD management.^5^

## Challenges and Potential Downsides

1. Risk of incentivizing marginal practices: Tying financial rewards to transplant volume may encourage accepting marginal kidneys for marginal recipients, leading to inferior outcomes and undermining long-term goals.^6^
2. Effect on resource allocation: The finite nature of transplant funding requires careful allocation to balance competing priorities. Reallocating resources to reduce kidney discards, for example, may divert funds from other critical areas like improving organ recovery or supporting long-term post-transplant care. Trade-offs include potentially fewer investments in donor hospital partnerships or reduced support for patient education programs. High-risk transplants require significant resources, and programs with limited staff or experience may struggle to meet demands without compromising care quality.
3. Equity concerns: Although waitlist criteria transparency is positive, disparities in health care access, particularly in rural or underserved areas, may worsen inequities in transplantation access. While public disclosure of waitlist criteria increases accountability, it may also expose preexisting resource gaps (*e.g*., in rural or underserved areas, geographic isolation, and socioeconomic factors) that could worsen inequities if not addressed. However, this transparency also opens avenues for advocacy and policy reform, as patients and stakeholders can better identify and challenge disparities.
4. Potential for gaming the system: Programs may prioritize low-risk patients to maximize financial gain, reducing access for high-risk patients and undermining the model's intent. Transparent communication with transplant candidates is essential to ensure they understand how financial incentives might influence decision making, preserving trust and ethical integrity in the transplant process.
5. Exclusion of transparency in organ offer declines: Omission of retrospective transparency on declined organ offers limits opportunities to address inefficiencies and improve organ allocation.
6. Fixed downside payment: A maximum downside payment of $2000 may not sufficiently deter suboptimal practices, reducing accountability for poor outcomes.
7. Disproportionate weighting of score points: The IOTA model places too much emphasis on achievement (60 points), which may encourage focusing on transplant numbers over efficiency and long-term quality.
8. Deceased donor kidney discards: High discard rates of deceased donor kidneys limit transplant growth, with the opioid-driven donor pool plateauing, stressing the need to optimize organ use.

## Balancing Trade-Offs

1. Defining success beyond transplant rates: Success must include quality indicators such as graft survival, patient-reported outcomes, and health care equity, not just transplant volume.
2. Investing in infrastructure and training: Managing complex cases requires workforce training, interdisciplinary collaboration, and patient education, with federal or state support ensuring equity for resource-limited centers.
3. Monitoring and mitigating unintended consequences: CMS should prevent system gaming, such as favoring healthier candidates, through oversight, patient advocacy groups, audits, and stakeholder feedback.
4. Incorporating data transparency: While organ-offer transparency was excluded, CMS should revisit this by sharing anonymized data to improve processes and accountability.
5. Addressing disparities: CMS must distribute resources equitably, supporting underperforming programs in rural or underserved areas to meet the model's goals.
6. Using evolving metrics to measure true program success presents inherent challenges, particularly in distinguishing the effect of Organ Procurement Organization procurement practices from center-level improvements. This complexity can obscure the root causes of performance changes, making it difficult to assess progress accurately. In addition, the lack of additional funding for staffing and infrastructure may strain resources, further complicating efforts to meet evolving benchmarks and achieve meaningful improvements in transplant outcomes.

## Recommendations for Stakeholders

### Recommendations for Centers

1. Rely on evidence-based decision making: Centers should use robust clinical evidence for donor-recipient matching, prioritizing long-term over short-term outcomes.
2. Invest in infrastructure and training: Strengthen data collection, care coordination, and patient education while training staff on new metrics and practices.
3. Adopt ethical practices to avoid gaming the system, such as equitable patient selection.
4. Focus on improving performance metrics while maintaining high-quality care.
5. Enhance patient engagement: Transparent communication on program participation and criteria builds trust and supports shared decision making
6. Address living donor outcomes: Centers must prioritize long-term health of living donors, especially those with multiple relative contraindications.

### Recommendations for CMS

1. Establish ongoing monitoring and adaptive policy adjustments to ensure the IOTA model's integrity.
2. Facilitate standardized public reporting to enhance transparency and accountability.
3. Strengthen oversight of combined organ transplant criteria: Implement clear monitoring to track safety-net transplant rates, prevent artificial number inflation, and maintain equity and quality.

#### Communication Roles

1. Centers must openly discuss financial incentives with patients to ensure informed consent.
2. CMS should communicate policy changes and performance benchmarks clearly to all stakeholders to maintain trust and alignment.
3. Monitor unintended consequences: CMS and programs must address issues such as allocation disparities or declines in outcome quality.

## Opportunities for Innovation

1. Promoting highly effective organ allocation by integrating advancing artificial intelligence in transplantation: AI-driven tools could enhance donor-recipient matching, predict outcomes, and optimize organ allocation.^7,8^ The IOTA model's emphasis on innovation and outcomes provides a unique testbed for implementing and validating these technologies, particularly in addressing historical allocation inefficiencies and disparities.
2. Promoting collaborative care models: The model's focus on long-term outcomes encourages collaboration among nephrologists, transplant surgeons, and primary care providers. This interdisciplinary approach is crucial for managing complex cases, particularly during high-risk periods such as prolonged delayed graft function or those in critical transition periods immediately post-transplant, at 1 year, and at 3 years, as defined by center-specific criteria. Such an approach ensures continuity of care and promotes optimized long-term outcomes.^9^
3. Promoting patient-centered care: Transparency and accountability foster trust and empower patients to actively participate in their care. Programs could develop educational initiatives to help patients understand their options and navigate the transplant process.
4. Kidney transplantation is at the core of all organ transplants: The IOTA model can serve as both a plea and a reference point—offering valuable lessons from its successes and helping to avoid potential pitfalls as it is explored for other organ transplants in the future.
5. Establishing a mechanism for independent oversight and auditing to assess the impact of policy changes: This raises the critical question: “*Who will watch the watchers?*” Given the significance of such a shift, it is imperative to involve an independent panel comprising nephrologists, patients, and laypeople. This panel should be structured with clear selection criteria, well-defined roles, and established mechanisms for ongoing feedback, ensuring that patients and donors play a tangible and integral role in the monitoring process. Such a panel could provide unbiased evaluations of the model's outcomes, ensuring transparency, accountability, and alignment with the goals of equity and improved patient care in transplantation. Independent panels should include structured roles for patient and donor representatives, such as serving on advisory boards or participating in audits. For example, patient advocacy organizations could nominate delegates to review outcome data and policy adjustments, ensuring lived experiences inform oversight. CMS could formalize this through grant-funded patient liaison programs.

## Conclusion

The IOTA model, approved by the Biden-Harris administration and facilitated by CMS, aims to transform kidney transplantation in the United States by increasing access and improving outcomes. However, it faces complex challenges in balancing volume incentives with quality care and system gaming, particularly regarding resource allocation and equity. Its success will depend on aligning financial incentives with quality and equity to improve patient outcomes. Ongoing monitoring and adaptive policy adjustments will be essential to ensure the IOTA model achieves its goals—improving transplant access and outcomes—while mitigating unintended consequences, such as inequitable patient selection or resource misallocation. Continuous evaluation and refinement will be critical to maintaining the model's integrity and effectiveness. By addressing these challenges and leveraging innovations such as AI-driven matching, collaborative care, and transparent patient engagement, the IOTA model could set a global benchmark for transplantation reforms. Stakeholders must ensure its implementation achieves these goals without compromising equity or patient outcomes.
